# Emerging roles of ferroptosis-related miRNAs in tumor metastasis

**DOI:** 10.1038/s41420-023-01486-y

**Published:** 2023-06-27

**Authors:** Zhongyi Jiang, Jing Zhou, Junqi Deng, Luohong Li, Ruifeng Wang, Yingying Han, Junyu Zhou, Rui Tao, Lushan Peng, Dan Wang, Tao Huang, Yupei Yu, Zongjiang Zhou, Jinghe Li, Diabate Ousmane, Junpu Wang

**Affiliations:** 1 Department of Pathology, Xiang-ya Hospital, Central South University, Changsha City, Hunan Province China; 2 Department of Pathology, School of Basic Medicine, Central South University, Changsha City, Hunan Province China; 3 Ultrapathology (Biomedical electron microscopy) Center, Department of Pathology, Xiang-ya Hospital, Central South University, Changsha City, Hunan Province China; 4 Key Laboratory of Hunan Province in Neurodegenerative Disorders, Xiangya Hospital, Central South University, Changsha, Hunan China; 5 National Clinical Research Center for Geriatric Disorders, Xiangya Hospital, Central South University, Changsha, China

**Keywords:** Cell death, Cancer microenvironment, Metastasis

## Abstract

Ferroptosis, a novel mode of cell death dependent on iron and reactive oxygen species, has been extensively explored during malignant tumors metastasis. Ferroptosis can interact with multiple components of the tumor microenvironment to regulate metastasis. These interactions generally include the following aspects: (1) Epithelial-mesenchymal transformation, which can help cancer cells increase their sensitivity to ferroptosis while they have multiple mechanisms to fight against it; (2) Disorder of iron metabolism in cancer stem cells which maintains their stem characteristics; (3) Polarization of M0 macrophages to M2. (4) The paradoxical effects of iron metabolism and CD8 + T cells induced by ferroptosis (5) Regulation of angiogenesis. In addition, ferroptosis can be regulated by miRNAs through the reprogramming of various intracellular metabolism processes, including the regulation of the glutathione- glutathione peroxidase 4 pathway, glutamic acid/cystine transport, iron metabolism, lipid metabolism, and oxidative stress. Therefore, there are many potential interactions between ferroptosis-related miRNAs and tumor metastasis, including interaction with cancer cells and immune cells, regulating cytokines, and angiogenesis. This review focuses on the role of ferroptosis-related miRNA in tumor metastasis, aiming to help readers understand their relationship and provide a new perspective on the potential treatment strategies of malignant tumors.

## Facts

1. Ferroptosis can interact with multiple components of the tumor microenvironment to regulate metastasis.
2. miRNAs can influence the progression of ferroptosis through different pathways.
3. There is a potential link between miRNA, ferroptosis and tumor metastasis.
4. Ferroptosis-related miRNAs are used as biomarkers for the diagnosis and prediction of tumor diseases.

## Open question

1. How does ferroptosis-related miRNA affect tumor progression, especially tumor metastasis?
2. Can ferroptosis-related miRNA be used for targeted therapy of tumor diseases?
3. Can we summarize the association of ferroptosis-related miRNAs with tumor metastasis through specific mechanisms?

## Introduction

Ferroptosis was first discovered in 2012 and has widely attracted attention as a novel mode of cell death. Pathological characteristics of ferroptosis are mainly reflected in mitochondria, including smaller size, reduced or disappeared cristae, membrane concentration, and outer membrane rupture [1–3]. On the molecular level, ferroptosis needs to be triggered by intracellular iron overload and reactive oxygen species (ROS) production and accumulation [4].

The relationship between ferroptosis and tumor metastasis has received extensive attention. At present, studies have found lipid peroxidation and iron overload in cancer cells [5]. Theoretically, ferroptosis is an anti-tumor mechanism. However, the current research has found that cancer cells can escape from ferroptosis through a variety of mechanisms, which is conducive to the progression and metastasis of cancer [6].

MicroRNAs (miRNAs) are small, non-coding RNAs that downregulate gene expression by targeting the 3’UTR of mRNA during transcription [7]. Notably, studies have found that miRNA can also regulate ferroptosis by combining with mRNA [8, 9]. The effects of miRNAs include targeting glutamate/cystine transport to regulate glutathione synthesis [10], iron metabolism to regulate intracellular iron content, lipid metabolism to regulate reactive oxygen species (ROS) generation, and other oxidative stress pathways [11]. Ferroptosis-associated miRNA has multiple roles in the process of tumor metastasis, including regulating tumor cells, immune cells, angiogenesis, and cytokine secretion, etc. [12].

In this review, we discussed the relationship between ferroptosis and tumor metastasis, analyzed the mechanisms of miRNA regulating ferroptosis, and finally summarized the new roles of ferroptosis-related miRNAs in tumor metastasis. We hope this review could provide new suggestions and references for future research, and the treatment of malignant cancer.

## Ferroptosis and metastasis in the tumor microenvironment (TME)

Metastasis is one of the prominent features of malignant tumors. It mainly contains the following steps. First, cancer in situ grows larger, and then invades the local blood vessels, lymph duct, or spreads directly. Then cancer cells can move with lymph or blood, gathering, adhering, and proliferating in the appropriate pre-metastatic niche of the secondary site where the structure of vessels or lymphatic ducts in the secondary site is changed and their permeability is increased, allowing the cancer cells to penetrate out of the circulatory system to invade and proliferate [13–15]. Recent studies have found that ferroptosis plays a regulatory role in the metastasis of malignant tumors, which mainly includes the regulation of tumor cells, cancer stem cells (CSCs), immune cells, epithelial-mesenchymal transition (EMT), tumor angiogenesis and so on.

### Ferroptosis and cancer cells

**Fig. 1:**
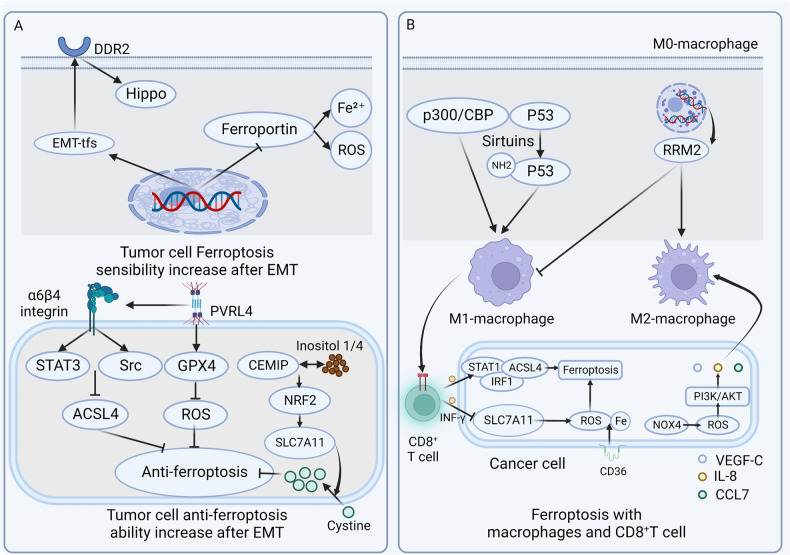
Ferroptosis with EMT and immune cells in the TME. **A** Some EMT-TFS can induce the expression of DDR2 to increase the ferroptosis sensitivity of cancer cells after EMT through the Hippo pathway, and inhibit the expression of iron export proteins to maintain intracellular iron overload and higher levels of ROS. The α6β4 integrin located on the cell membrane can activate the Src and STAT3 pathways to inhibit the expression of the long polyunsaturated fatty acid-rich enzyme ACSL4. The adhesion protein PVRL4 inhibits ferroptosis through the PVRL4/α6β4/Src signaling pathway. CEMIP interacts with inositol 1/4 to activate NRF2 to increase SLC7A11 transcription, and cancer cells uptake cystine to increase ferroptosis resistance after ECM detachment. **B** Iron overload can increase intracellular ROS, and activate macrophages to M1 phenotype by enhancing P300 /CBP acetyltransferase activity and promoting p53 acetylation, thereby promoting the progression of inflammation. At the same time, RRM2 gene expression can inhibit ferroptosis, promote the polarization of M2 macrophages, and inhibit the polarization of M1 macrophages to promote cancer progression and metastasis. The increased expression of NOX4 can activate the PI3K/Akt signaling pathway through the increase of ROS, causing the increased secretion of various cytokines such as VEGF-C, IL-8, and CCL7, and promoting the polarization of M2 phenotype macrophages. In cancer cells, M1 macrophages can activate CD8 + T cells to cause ferroptosis and play a killing role. CD8 + T cells downregulate the expression of SLC3A2 and SLC7A11, two subunits of XC—by releasing INFγ, which affects the uptake of cystine and promotes the production of ROS and ferroptosis. INFγ could also activate ACSL4 expression through the STAT1-IRF1 signaling pathway and trigger ACSL4-dependent ferroptosis. Cholesterol can increase the expression of CD36 in CD8 + T cells, increase the uptake of fatty acids by T cells, induce lipid peroxidation and ferroptosis of CD8 + T cells, and cause tumor-killing ability dysfunction.

Cancer in situ show the resistance to ferroptosis due to the high cell density and tight cell junctions. For most malignant tumors, EMT is the first procedure for the metastasis. Studies have found that cancer cells are connected by E-cadherin (ECAD), which is also increased in tumors with high cell density, inhibiting ferroptosis through the Ecad-NF2-Hippo-YAP signaling axis. After EMT, the reduction of ECAD could cause the increased sensitivity to ferroptosis [16–18]. Some studies used histone deacetylase (HDAC) inhibitors to induce EMT of human adrenal cortical cancer. In this process, the decreased expression of iron-exporting protein and antioxidant genes in cancer cells resulted in increased intracellular iron accumulation and the expression of ROS which indicates that cancer cells going through EMT have a greater possibility and sensitivity to ferroptosis [18]. Some other studies have found that some EMT transcription factors (EMT-TFs) (TWIST/SNAIL) can induce discoidin domain receptor2 (DDR2) expression thus increasing the sensitivity to ferroptosis through the Hippo pathway [19]. Cancer cells have an increased sensitivity to ferroptosis after EMT, which appears to be detrimental to tumor metastasis. In fact, they also have a variety of mechanisms to combat ferroptosis. It is reported that α6β4 integrin is involved in the anti-ferroptosis process [20, 21]. α6β4 integrin is an important component of type I hemidesmosomes (HD), which is found in the epithelium of various tissue types and participating in initiating HD assembly and mediating cell adhesion [22]. Once detached from ECM, α6β4 integrin on cancer cells inhibited the expression of long polyunsaturated fatty acid-rich enzyme acyl-CoA synthetase long-chain family member 4 (ACSL4) (essential for ferroptosis) through Src and signal transducer and activator of transcription 3 (STAT3) pathways [20]. The adhesion protein poliovirus receptor-related protein 4 (PVRL4) could also participate in this process which facilitate the accumulation of cancer cells isolated from ECM spontaneously and inhibit ferroptosis through PVRL4/α6β4/Src signaling pathway [21]. It also stimulates and maintains the expression of glutathione peroxidase 4 (GPX4), which can convert lipid hydroperoxides to lipid alcohols and inhibit the formation of ROS, thereby inhibiting ferroptosis [23]. In recent studies, it has been found that the interaction of cell migration-inducing protein (CEMIP) with inositol 1/4 in prostate cancer cells activates the nuclear factor erythroid 2-related factor 2 (NRF2) and promotes the transcription of solute Carrier Family 7, Member 11 (SLC7A11), (a glutamate/cysteine antiporter solute family 7 member). Promoting cystine uptake by cancer cells could increase ferroptosis resistance after ECM detachment [24]. This sensitization to ferroptosis after EMT and the anti-ferroptosis effect of cancer cells is a contradictory relationship that results in the release of large numbers of cancer cells from the carcinoma in situ tissue, but only some of them could successfully colonize (Fig. 1A).

CSCs exist in the primary tumor site of the TME, which are associated with strong differentiation and proliferation ability, and they are considered to be critical for metastasis [25]. Some stem cell markers and signaling pathways in CSCs are also related to iron, and the high density of intracellular iron is involved in maintaining CSC stem characteristics, protecting [26, 27]. The phenotype of CSCs is plastic and can be influenced by various signals in the TME, such as the Wnt signal and nuclear factor kappa-B (NF-κB) signal to transform between stem cell type and non-stem cell type [25]. Recently, the phenotypic plasticity of CSCs has been found to protect metastatic breast cancer cells from ferroptosis. Screening the secretome of breast cancer stem cells (BCSCs) revealed that dickkopf-related protein 1 (DKK1) reduced the stem cell phenotype by inhibiting Wnt signaling in metastatic cancer cells. DKK1 also reduced SLC7A11 expression and lipid peroxidation, and increased glutathione, thereby reducing ferroptosis [28].

### Ferroptosis and immune cells

#### Ferroptosis and tumor-associated macrophages (TAMs)

TAMs are one of the most significant regulatory cells in the TME. Among them, M2-phenotype macrophages account for the majority in the late stage of malignant tumors, which promote tumor progression and angiogenesis, mediating malignant tumor immunosuppression, and metastasis [29, 30]. Studies found that there are many types of receptors on the surface of TAMs, which can trigger ferroptosis of tumor cells through a variety of signaling pathways, and affect the polarization state of macrophages [31]. For instance, iron overload can increase the expression of ROS in macrophages. Then, they are activated to the M1 phenotype by enhancing P300/cAMP-response element binding protein (CREB)-binding protein (CBP) acetyltransferase activity and promoting p53 acetylation, thereby improving the progression of inflammation [32]. In cancer cells, M1 macrophages are the main cause of ferroptosis through the activation of CTL.

At late stages of cancer, macrophages with M2 phenotype are increased. Ribonucleoside-Diphosphate reductase subunit M2 (RRM2), one of the regulatory factors related to ferroptosis, affects the prognosis of lung adenocarcinoma through database analysis. Subsequent experiments showed that RRM2 could inhibit ferroptosis, promote the polarization of M2 macrophages, and inhibit the polarization of M1 macrophages to facilitate cancer progression and metastasis [33, 34]. In non-small cell lung cancer, NADPH oxidase 4 (NOX4) expression is increased, which can activate the PI3K/Akt signaling pathway through ROS increase. It upregulates the secretion of various cytokines such as vascular endothelial growth factor-C (VEGF-C), IL-8, and chemokine (C-C motif) ligand 7 (CCL7), which promote M2 macrophage polarization and metastasis (Fig. 1B) [35].

At present, the direct link between ferroptosis and TAM as well as the related mechanisms and pathways are still not clear. However, it is undeniable that clarifying the relationship is promising for the treatment. Studies have used nanomaterials equipped with ferroptosis inducers to increase the anti-inflammatory response of tumor cells through ferroptosis stress, induce the polarization of M1 phenotype macrophages, and regulate inflammatory and metabolic functions for tumor treatment [36].

#### Ferroptosis and T cell

Ferroptosis also interacts with various immune cells in the TME, and the relationship with CD8 + T cells has been most deeply explored. The traditional view is that CD8 + T cells kill tumor cells through perforin-granzyme and Fas-Fasl mechanisms [37–39]. With a deeper understanding of ferroptosis, researchers found that CD8 + T cells can also induce cancer cells ferroptosis. CD8 + T cells downregulate the expression of SLC3A2 and SLC7A11, two subunits of the glutamate-cystine antiporter system Xc- by releasing interferon-γ (INF-γ), which affects cystine uptake and promotes ROS production and ferroptosis [38]. Recent studies have found that INF-γ could activate ACSL4 expression through the signal transducer and activator of transcription 1 -interferon regulatory factor 1 (STAT1-IRF1) signaling pathway, leading to ACSL4-dependent ferroptosis [38, 40]. However, this killing effect could not be maintained for long, after which cholesterol in the TME could induce CD36 expression on CD8 + T cells. This increases the uptake of fatty acids by T cells, resulting lipid peroxidation, and ferroptosis of CD8 + T cells themselves. Leading the loss of tumor-killing function and anti-tumor ability gradually, thus increasing tumor metastasis (Fig. 1B) [41–43].

#### Ferroptosis and other immune cells

The TME can cause metabolic reprogramming in other different types of immune cells, including abnormal iron metabolism and lipid peroxidation. But there are few studies on the direct relationship between them. At present, omics studies have found that the ferroptosis regulatory gene SLC7A11 can reduce the abundance of NK cells and inhibit anti-tumor immunity [44]. Experiments in vitro revealed that the mitochondrial iron transport protein ATP-binding cassette transporter 7 (ABCB7) is essential for B cell development, proliferation, class switching, and recombination [45]. Minghua Yang et al. discovered that granulocytes in glioblastoma can trigger cancer cell ferroptosis. Neutrophils can transfer myeloperoxidase into cancer cells, inducing iron-dependent accumulation of lipid peroxides [46]. The interaction between ferroptosis and immune cells needs more deeper research to analyze the mechanism, pathway, and effect on tumor progression and metastasis.

### Ferroptosis and angiogenesis

Angiogenesis is one of the significant features of malignant tumors. Although the direct role of ferroptosis in this process has not been fully investigated, ROS production has been shown to participate in tumor angiogenesis [47]. In endothelial cells, ROS can be derived from NADPH oxidase in mitochondria. Under the stimulation of inflammation and tumor substances, it can cause the expression of angiogenic cytokines such as VEGF. Activated NOX and ROS could change local vascular endothelial cells from a static to a proliferative state, which is conducive to angiogenesis [48]. At present, the relationship between ferroptosis and angiogenesis is not clear. Recent studies have reported that miR-539 can activate the stress-activated protein kinase/jun N-terminal kinases (SAPK/JNK) signaling pathway by targeting tumor necrosis factor (TNF) -α-induced protein 8 (TIPE) and reduce the expression of GPX4 to inhibit ferroptosis in colorectal cancer cells [49]. Meanwhile, TIPE promotes vascular endothelial growth factor receptor 2 (VEGFR2)-mediated angiogenesis by upregulating the expression and phosphorylation of pyruvate dehydrogenase kinase 1 (PDK1) in cancer cells [50], thus promoting metastasis.

## miRNAs and ferroptosis

The emerging evidence suggests that miRNAs are involved in regulating multiple key steps of ferroptosis, including glutathione-GPX4 pathway, glutamate/cystine transport, iron metabolism, and lipid metabolism.

### miRNAs regulate the glutathione-GPX4 pathway

GPX4 is a glutathione-dependent enzyme that transforms toxic lipid hydroperoxides to nontoxic lipoalcohol (L-OH), thereby reducing the conversion of iron-induced lipid hydroperoxides to highly reactive lipid alkoxyl radicals, and restraining ferroptosis. The inhibition of GPX4 promotes the progression of ferroptosis. For example, miR-15 can inhibit GPX4 expression by interacting with the 3’-UTR of GPX4 mRNA [51], which blocks the conversion of glutathione (GSH) to L-glutathione Oxidized (GSSG), hinders the conversion of hazardous lipid peroxides into nontoxic L-OH, increases GSH, ROS levels and malondialdehyde (MDA), and accelerates the progress of ferroptosis. miR-15a-3p [52], miR-1287-5p [53], and miR-324-3p have similar roles in cancer by directly targeting GPX4 to positively regulate ferroptosis [54].

What’s more, activating transcription factor 4 (ATF4) is a member of the CREB / ATF family, and acts as a transcriptional activator and repressor in ferroptosis. MicroRNA-214-3p plays a regulatory role in hepatocarcinogenesis, and the inhibition of miR-214 can directly promote the expression of ATF4 [55]. ATF4 binds with an amino acid reaction element in the promoter region of the SLC7A11, promoting the transcription of SLC7A11. On the other hand, ATF4, a key point of endoplasmic reticulum stress affecting ferroptosis, promotes heat shock 70 kDa protein 5 (HSPA5) expression through the PERK-ATF4-CHOP pathway, increases GPX4 expression, hinders ROS generation, and suppresses ferroptosis.

### miRNAs regulate glutamate/cystine transport

It has been confirmed that the regulation of the glutamate/cysteine reverse transport system key proteins can affect ferroptosis in tumor cells, such as SLC38A1, SLC1A5, SLC3A2, and SLC7A11. System Xc-(SLC3A2 and SLC7A11) pumps glutamate away from the cell in a 1:1 ratio while transferring the extracellular cystine to the cell [56]. When System Xc^-^function is inhibited or deactivated, it leads to cysteine depletion, lipid peroxidation, and ferroptosis [10]. miR-5096 [57], miR-27a-3p [58], miR-375 [59], and miR-34c-3p can inhibit cysteine and GSH generation by directly targeting SLC7A11 [60], which increase ROS and iron accumulation levels, thus promoting ferroptosis. Acting as a chaperone of SLC7A11, SLC3A2 contributes to the stability of SLC7A11 protein. miR-142-3p has been found to highly express in the M1 macrophages of hepatocarcinoma tissue, which could affect the production of GSH, Fe^2+^, and MDA, promoting ferroptosis of the M1 macrophages by targeting SLC3A2, thus facilitating tumor metastasis [61]. SLC1A5 and SLC38A1 can mediate the uptake of neutral amino acids such as glutamine (Gln). Direct targeting of SLC1A5 to inhibit Gln uptake by miR-137 resulted in a decrease in the generation of glutamate (Glu) and ROS, thereby inhibiting ferroptosis. Although α -ketoglutarate (α-KG) can enhance insensitive ferroptosis of miR-137 to rescue the overall inhibitory effect, it also suggests that SLC1A5 is located upstream in the Gln decomposition process [62].

Furthermore, β-mercaptoethanol can convert the incoming cystine into cysteine without relying on systemic XC^-^. Cystethiane β-synthase (CBS) acts as a marker of transsulfuration activity, participates in ferroptosis and protects hepatocellular carcinoma, lung cancer, and breast cancer cells from ferroptosis [63]. miR-6852 can directly bind to CBS, reduce cysteine uptake, increase the intracellular concentrations of lipid ROS, reduce the cellular mitochondrial membrane potential and promote ferroptosis [63].

### miRNAs regulate iron metabolism

Excessive iron accumulation is the basis of ferroptosis. Iron bind to transferrin and exist in the form of Fe^3+^, after entering the cells through the transferrin receptor (TFR1) on the cell membrane, it is converted to the Fe^2+^ under the function of six-transmembrane epithelial antigen of the prostate 3 (STEAP3) [64]. Later, the divalent metal ion transporter 1 (DMT1) transports it to the cytoplasm of the unstable iron pool. The intracellular accumulation of excessive Fe^2+^ can trigger Fenton reactions to generate Fe and hydroxyl radicals, thus producing a large amount of ROS. It has shown that miR-148a [65], miR-107 [66], and miR-378g can downregulate TFR1 expression by binding to TFR1 3’UTR [67], affecting intracellular iron homeostasis and promoting the proliferation of hepatocarcinoma (HCC), colorectal cancer, and laryngeal cancer cells, respectively.

Excessive intracellular Fe^3+^ can be stored in ferritin, and the unbound part is excreted outside by membrane ferroportin 1 (FPN1), thereby maintaining intracellular iron metabolism homeostasis. Ferritin heavy chain 1 (FTH1) and ferritin light chain (FTL) both are the components of ferritin [68]. It has been shown that lncRNA H19 can enhance the transcriptional activity of the endogenous FTH1 by sponging miRNA-19b-3p, which increases the storage of ferric ions to reduce the intracellular free iron content and inhibits ROS generation and ferroptosis [69]. miR-147a [70], miR-302a-3p [71], and miR-153-5p can directly bind to the 3’-untranslated region of FPN1 and inhibit iron export, thereby promoting iron overload, lipid peroxidation, and ferroptosis [72]. What’s more, Nrf2 can increase the transcription level of FPN1, thereby increasing the extracellular output of intracellular iron and reducing the Fe^2+^ levels in the intracellular labile iron pool (LIP). In myeloma cells, Nrf2 directly trans-activates FPN1 or promotes FPN1 expression by inhibiting miR-17-5p, promoting iron export, reducing the intracellular ferric ion concentration and ROS production, and inhibiting ferroptosis [73]. This provides a new direction to target the iron mechanism.

### miRNAs regulate lipid metabolism

One of the characteristics of ferroptosis is the accumulation of ROS through polyunsaturated fatty acid (PUFA) peroxidation [74]. For example, arachidonic acid (AA) and adrenal acid (AdA) are the main free PUFAs, and ACSL4 uses coenzyme A to catalyze the AA/AdA for the generation of AA / AdA-CoA, then esterifying to AA/AdA-PE under the influence of lysophosphatidylcholine acyltransferase 3 (LPCAT3), and finally oxidized to lipid peroxides by ALOXs. The downregulated miR-424-5p can directly bind to the 3’-UTR of ACSL4 to improve the expression of ACSL4 [75], promote the accumulation of lipid peroxides, destroy the integrity of cell membrane and the release of cell contents, which triggers ferroptosis [76]. Similarly, the downregulation of miR-670-3p [77] and miR-23a-3p also accelerate ferroptosis by targeting ACSL4 [78]. Cisplatin and paclitaxel can increase miR-522 secretion of fibroblast exosomes by activating the USP7/hnRNPA1 axis, while miR-522 can directly interact with arachidonate 15-lipoxygenase (ALOX15), reduce the conversion of PUFA to PUFAs-OOH, suppress lipid peroxidation and ROS production, and ultimately reduce chemosensitivity [79].

**Fig. 2:**
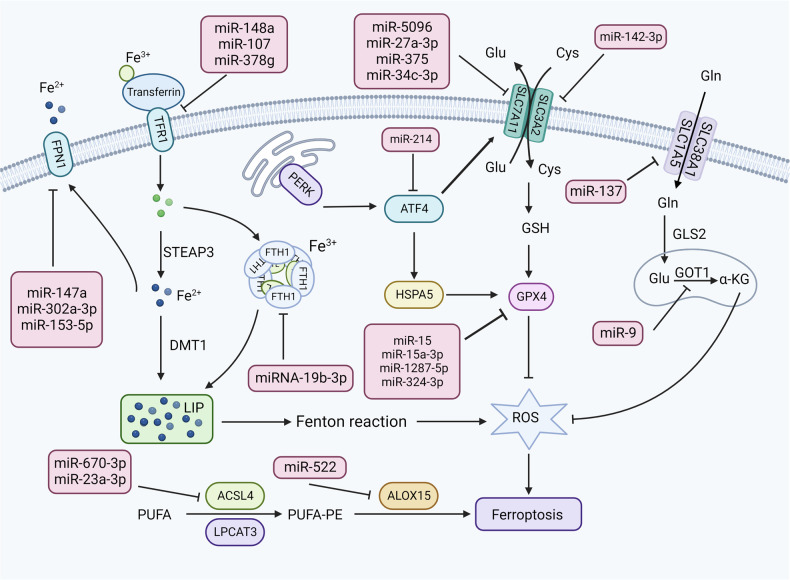
The mechanisms of miRNA regulating ferroptosis. miRNAs regulate the glutathione-GPX4 pathway, glutamate/cystine transport, iron metabolism, lipid metabolism, and other oxidative stress pathways to control ferroptosis.

Tumor cells prefer to synthesize fatty acids de novo than normal cells that tend to uptake them from exogenous sources. When lipid synthesis increases in cancer cells, more lipid biosynthetic enzymes are needed to produce different fatty acids [11]. Stearyl-coenzyme A desaturase 1 (SCD1) is an essential enzyme for the de novo synthesis of FA, which catalyzes the desaturation of saturated fatty acid to monounsaturated fatty acid (MUFA). LINC01606 promotes SCD1 expression and functions through interaction with miR-423-5p to control intracellular MUFA synthesis and activate Wnt/β-catenin signaling [80]. This signaling enhances LINC01606 expression to promote MUFA synthesis continuously, while MUFA competitively affects PUFA peroxidation [56], thereby inhibiting ferroptosis. (Fig. 2 and Table 1).

**Table 1:** Ferroptosis-related miRNAs in cancer.

miRNA	Mechanism	Function	Disease	References
miR-15a-3p	Downregulate GPX4	Induce ferroptosis	Colorectal cancer	[52]
miR-1287-5p	Downregulate GPX4	Induce ferroptosis	Osteosarcoma	[53]
miR-324-3p	Downregulate GPX4	Induce ferroptosis	Lung adenocarcinoma	[54]
miR-214	Downregulate ATF4	Induce ferroptosis	Hepatoma	[55]
miR-5096	Downregulate SLC7A11	Induce ferroptosis	Breast cancer	[57]
miR-27a-3p	Downregulate SLC7A11	Induce ferroptosis	NSCLC	[58]
miR-375	Downregulate SLC7A11	Induce ferroptosis	Gastric cancer	[59]
miR-34c-3p	Downregulate SLC7A11	Induce ferroptosis	Oral squamous cell carcinoma	[60]
miR-142-3p	Downregulate SLC3A2	Induce ferroptosis	Hepatocellular carcinoma	[61]
miR-137	Downregulate SLC1A5	Repress ferroptosis	Melanoma	[62]
miR-9	Downregulate GOT1	Repress ferroptosis	Melanoma	[111]
miR-6852	Downregulate CBS	Induce ferroptosis	Lung cancer	[63]
miR-19b-3p	Downregulate FTH1	Induce ferroptosis	Lung cancer	[69]
miR-147a	Downregulate FPN1	Induce ferroptosis	Prostate cancer	[70]
miR-302a-3p	Downregulate FPN1	Induce ferroptosis	NSCLC	[71]
miR-153-5p	Downregulate FPN1	Induce ferroptosis	Renal cell carcinoma	[72]
miR-17-5p	Downregulate FPN1	Induce ferroptosis	Multiple myeloma	[73]
miR-130b-3p	Downregulate DKK1	Repress ferroptosis	Melanoma	[112]
miR-19a	Downregulate IREB2	Repress ferroptosis	Colorectal cancer	[113]
miR-129-5p	Downregulate PROM2	Induce ferroptosis	Bladder cancer	[114]
miR-424-5p	Downregulate ACSL4	Repress ferroptosis	Ovarian cancer	[75]
miR-670-3p	Downregulate ACSL4	Repress ferroptosis	Glioblastoma	[77]
miR-23a-3p	Downregulate ACSL4	Repress ferroptosis	Hepatocellular carcinoma	[78]
miR-522	Upregulate Alox15	Induce ferroptosis	Gastric cancer.	[79]
miR-423-5p	Downregulate SCD1	Induce ferroptosis	Colon cancer	[80]
miR-4443	Upregulate FSP1	Repress ferroptosis	NSCLC	[115]
miR-101-3p	Downregulate CISD1	Induce ferroptosis	Lung adenocarcinoma	[116]
miR-6077	Downregulate Keap1	Repress ferroptosis	Lung adenocarcinoma	[117]

miRNAs regulate ferroptosis by targeting different genes in the glutathione-GPX4 pathway, glutamate/cystine transport, iron metabolism, lipid metabolism, and other oxidative stress pathways to control ferroptosis.

## The role of the ferroptosis-related miRNAs in tumor metastasis

Ferroptosis can play a regulatory role in the process of tumor metastasis, including regulating tumor cells, tumor stem cells, immune-related cells, tumor angiogenesis and so on. Considering that miRNAs could participate in the process of ferroptosis, the ferroptosis-related miRNAs could affect tumor metastasis, as well.

### Ferroptosis-related miRNAs regulate tumor cells

The miRNA can promote metastasis by regulating morphological transition. miR-15a-3p is significantly downregulated in ovarian cancer [81], gastric cancer, and small-cell lung cancer [82]. It directly boosts the expression of E-cadherin, N-cadherin, and c-fos through Twist1, which induces EMT and enhances tumor migration and metastasis [83]. Interestingly, Twist1 is an inhibitor of ATF4, which activates the ATF4/CHAC1 pathway to induce GSH degradation, affecting ferroptosis [84]. Moreover, miR-15a-3p can inhibit pancreatic cancer cell proliferation, invasion, EMT, and stemness characteristics by suppressing the SLC39A7-mediated Wnt/β-catenin pathway [85]. What’s more, Wnt/β-catenin pathway can inhibit PUFA peroxidation by enhancing MUFA, which is associated with the downregulation of ferroptosis [80], which is a disadvantage for ferroptosis. Notably, ferroptosis-associated miR-5096 can not only target SLC7A11 to increase ROS and iron accumulation directly, but also regulate EMT marker expression and inhibits the metastatic potential of the cells [57], thus hindering tumor metastasis.

CSCs are the source of cancer initiation, recurrence, and metastasis. The specific miRNAs have regulatory effects on CSCs. Moreover, EMT and CSC formation have a strong correlation with tumor invasion and metastasis, and they are associated with tumor cell chemoresistance [86]. The level of miR-214-3p is significantly low in LSCC tissues while the level of yes-associated protein 1 is high, and it maintains CSC properties by activating the Hippo signaling pathway [87]. While downregulation of YAP/TAZ activity after DDR2 knockdown may contribute to ferroptosis protection.

miRNA can regulate tumor metastasis through intercellular communication with the tumor cells. Exosomes are a subset of membrane-bound extracellular vesicles ranging from 50 to 150 nm in diameter and contain multiple molecules, such as miRNA, leading to the miRNA transition to other tumor cells through exosomes. miR-9 secreted by tumor cells can be transferred to normal fibroblasts through exosomes, thus improving cell motility in breast cancer by reducing the expression of E-cadherin and calcium-dependent cell-cell adhesion glycoproteins [88]. USP7 promotes the secretion of miR-522 derived from cancer-associated fibroblasts (CAFs) exosome by regulating hnRNPA1 deubiquitination, targeting ALOX15 in tumor cells to inhibit the production of ROS, and increasing the chemoresistance of gastric cancer cells [79].

### Ferroptosis-related miRNAs regulate immune cells

miRNA regulates the function of immune cells and tumor metastasis through several mechanisms, such as regulating metabolic reprogramming and macrophage phenotype. Leukemia cell-derived sEV-associated miR-19a-3p alters the metabolic reprogramming of CD8 + T cells by targeting SLC6A8-mediated creatine import, causing immune evasion and tumor metastasis [89]. CD8 + T cells are reduced by releasing IFN-γ, resulting in attenuated downregulation of SLC7A11. Besides, PD-L1 inhibits T-cell proliferation by binding to programmed death 1 (PD-1). It has been shown that ER-stressed HCC cells deliver miR-23a-3p in exosomes to macrophages, which activates the PI3K-AKT pathway by inhibiting phosphatase and tensin homolog (PTEN) to increase PD-L1 expression, reducing the CD8 + T cell ratio and IL-2 production, thereby resulting in tumor escape [90]. Similarly, miR-424-5p actively rescues the exhaustion of T cells by downregulating PD-L1 expression [91]. Furthermore, lncRNA FENDRR upregulates GADD45B to inhibit Treg-mediated immune escape of HCC cells by sponging miR-423-5p [92]. However, its effects on tumor metastasis meed to be further studied.

miRNA can promote tumor progression by regulating macrophage phenotype. miR-153-5p directly targets FPN1 to promote iron overload, lipid peroxidation, and ferroptosis. In addition, studies have shown that iron overload can increase the levels of M1 markers (e.g., IL-6, TNF-α, and IL-1β) and reduce M2 polarization, which restrains the tumor progression and metastasis [93]. Moreover, lncRNA FYVE, RhoGEF and PH domain containing 5 antisense RNA 1 (FGD5-AS1) upregulates bone marrow stromal cell antigen 2 (BST2) by sponging miR-129-5p, suppressing M1 macrophage polarization, and simultaneously inducing M2 macrophage polarization, thus increasing the development of cervical cancer [94]. A similar effect has been observed in miR-17-5p, whose over-expression could promote M2 polarization and enhance gastric cancer cell metastasis [95].

### Ferroptosis-related miRNAs regulate cytokines

Ferroptosis-related miRNA influences tumor metastasis by regulating the expression of cytokines (e.g., transforming growth factor-β 1 (TGF-β1), CCL5, and IL-8). TGF-β could regulate cell proliferation and differentiation through a series of signal pathways. In the SMAD pathway, TGF-β signaling activates drosophila mothers against decapentaplegic protein 2 (Smad2) and Smad3 via TβRI and TβRII to form the SMAD complex, which is transferred to the nuclear to promote EMT progression [96]. For example, miR-9 can directly target the 3’UTR of SOX7 and inhibit the transcriptional activity of SOX7 mRNA, thereby promoting TGF-β1-induced non small-cell lung cancer (NSCLC) cell metastasis [97]. miR-19a can induce EMT under the upregulation of TNF-α, thus promoting colorectal cancer (CRC) invasion and metastasis. However, the underlying molecular mechanisms are not fully cleared [98]. miR-129-5p could inhibit the metastasis of gastric cancer cells by downregulating IL-8 expression, which is known as the chemokine ligand (CXCL) 8 and plays a pivotal role in angiogenesis and metastasis [99].

### Ferroptosis-related miRNAs regulate angiogenesis

**Fig. 3:**
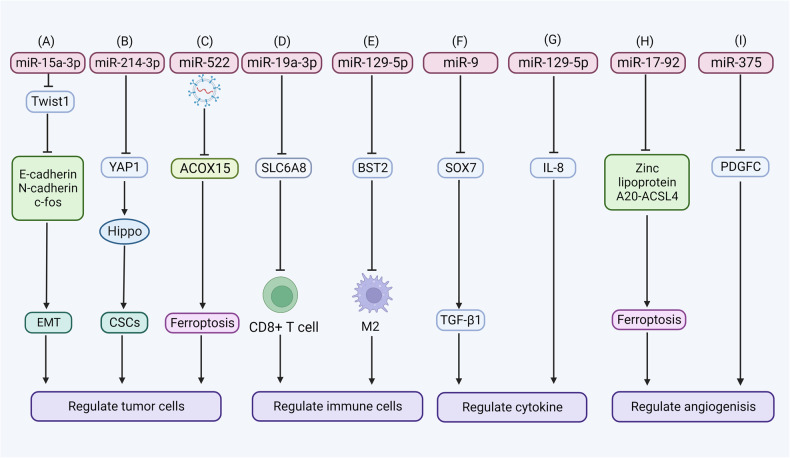
The mechanisms of ferroptosis-related miRNA regulating tumor metastasis. Ferroptosis-related miRNA relates to tumor metastasis by influencing tumor cells (EMT, CSCs, and exosome), immune cells, cytokines, and angiogenesis. **A** miR-15a-3p targets Twist1 to regulate E-cadherin, N-cadherin, and c-fos expression, which induces mesenchymal cell morphology and enhances tumor migration and metastasis. **B** miR-214-3p suppresses CSC by directly targeting the yes-associated protein 1 (YAP1) and activating the Hippo signaling pathway. **C** miR-522 derived from CAFs exosome targets ALOX15 in tumor cells to inhibit the production of ROS, and increasing the chemoresistance of cancer cells. **D** miR-19a-3p alters the metabolic reprogramming of CD8 + T cells by targeting SLC6A8-mediated creatine import, causing immune evasion. **E** miR-129-5p targets BST2 and suppress M2 macrophage polarization, thus increasing the development of cervical cancer. **F** miR-9 can directly target the 3’UTR of SOX7 and inhibit the transcriptional activity of SOX7 mRNA, thereby promoting TGF-β1-induced NSCLC cell metastasis. **G** miR-129-5p could inhibit the metastasis of gastric cancer cells by downregulating IL-8 expression. **H** miRNA-17-92 targets zinc lipoprotein A20-ACSL4 axis, protects endothelial cells from ferroptosis and facilitates angiogenesis. (**I**) miR-375 inhibits PDGFC and then inhibits the tumor angiogenesis.

Tumor angiogenesis could be regulated by ferroptosis-related miRNAs from different types of cells in the TME. miRNA-17-92, a miRNA cluster that promotes cancer progression, has been found to target zinc lipoprotein A20-ACSL4 axis, which protects endothelial cells from ferroptosis [100] and facilitates angiogenesis. LncRNA SNHG11 is highly expressed in pancreatic cancer patients, which promotes VEGFA expression by miR-324-3p, thus increasing tumor angiogenesis and facilitating metastasis [101]. In glioma, miR-9 is increased by the paracrine secretion of cancer cells. After being absorbed by endothelial cells, miR-9 acts on multiple targets and promotes angiogenesis [102]. Meanwhile, some ferroptosis-related miRNAs have an inhibitory effect on tumor angiogenesis. One study identified the role of miR-214 in inhibiting angiogenesis and promoting apoptosis in HCC by using a miRNAs mimetic and inhibitor transfection manner [103], while miR-214 is frequently downregulated in HCC, and the paracrine activation of hepatoma-derived growth factor (HDGF) promotes tumor angiogenesis. miR-375 inhibits the platelet-derived growth factor C (PDGFC) and then inhibits the tumor angiogenesis [104]. Therefore, ferroptosis-related miRNA can play a promoting or inhibitory role in the process of tumor angiogenesis, thus affecting the occurrence and development of tumor metastasis. (Fig. 3 and Table 2).

**Table 2:** The role of ferroptosis-related miRNAs in tumor metastasis.

Ferroptosis target	miRNA	Mechanism	Function	Disease	References
GPX4	miR-15a-3p	Downregulate Twist1	Regulate tumor cells	Ovarian cancer	[81]
GPX4	miR-324-3p	Downregulate WNT2B	Regulate tumor cells	Nasopharyngeal carcinoma	[118]
GPX4	miR-324-3p	Downregulate Smad4	Regulate tumor cells	Gastric cancer	[119]
GPX4	miR-214-3p	Downregulate YAP1	Regulate tumor cells	lung squamous cell cancer	[87]
ATF4	miR-214	Downregulate CTNNBIP1	Regulate tumor cells	Lung Adenocarcinoma	[120]
SLC1A5	miR-137	Downregulate KLF12	Regulate tumor cells	Hepatocellular carcinoma	[121]
GOT1	miR-9	Downregulate E-cadherin	Regulate tumor cells	Breast cancer	[88]
IREB2	miR-19a-3p	Downregulate SLC6A8	Regulate immune cells	Myeloid leukemia	[89]
ACSL4	miR-23a-3p	Downregulate PTEN	Regulate immune cells	Liver cancer	[90]
ACSL4	miR-424-5p	Downregulate PD-L1	Regulate immune cells	Breast cancer	[91]
SCD1	miR-423-5p	Downregulate GADD45B	Regulate immune cells	Hepatocellular carcinoma	[92]
PROM2	miR-129-5p	Downregulate BST2	Regulate immune cells	Cervical cancer	[94]
DKK1	miR-130b-3p	Downregulate DLL1	Regulate ECM	Breast carcinoma	[122]
ACSL4	miR-23a-3p	Downregulate HIF1-α/VEGF/MMP9	Regulate ECM	Glioblastoma	[123]
FSP1	miR-4443	Downregulate MMP-9	Regulate ECM	Melanoma	[124]
PROM2	miR-129-5p	Downregulate MMP-9	Regulate ECM	Melanoma	[125]
GOT1	miR-9	Upregulate TGF-β1	Regulate cytokine	Lung cancer	[97]
FPN1	miR-17-5p	Downregulate TGF-β	Regulate cytokine	CRC	[126]
FPN1	miR-147a	Downregulate CCL5	Regulate cytokine	NSCLC	[127]
PROM2	miR-129-5p	Downregulate IL-8	Regulate cytokine	Gastric cancer	[99]
FPN1	miR-17-5p	Downregulate CXCL14	Regulate cytokine	Glioma	[128]
ACSL4	miR-17-92	Downregulate GPX4	Regulate angiogenesis	-	[100]
GPX4	miR-324-3p	Upregulate VEGFA	Regulate angiogenesis	Pancreatic cancer	[101]
GOT1	miR-9	Upregulate HIF-1α/VEGF	Regulate angiogenesis	Glioma	[102]
ATF4	miR-214	Upregulate HDGF	Regulate angiogenesis	HCC	[103]
SLC7A11	miR-375	Downregulate PDGFC	Regulate angiogenesis	HCC	[104]

Ferroptosis-related miRNA relates tumor metastasis through influencing tumor cells (EMT, CSCs, and exosome), immune cells, cytokines, and angiogenesis.

## Application of ferroptosis-related miRNAs in tumor therapy

miRNAs could affect ferroptosis and regulate the metastatic process of tumors, which provides a new direction for the subsequent tumor diagnosis and drug development [105]. Currently, there are already ferroptosis-related miRNAs used as biomarkers for tumor diagnosis or prediction, like miR-214, miR-17-92, and miR-324-3p [106], etc. Moreover, targeted therapy of miRNAs in tumor metastasis is also under explored. Icariside II is a flavonoid with antitumor properties [107], which directly suppresses the GPX4 through miR-324-3p to induce ferroptosis, thus inhibiting the migration and invasion of renal cell carcinoma (RCC) [108]. This natural extract may be an effective drug for the treatment of RCC. As an analgesic, ketamine is ideal for managing cancer-related pain in clinical practice. Ketamine could significantly inhibit HCC progression by targeting lnvPVT1/miR-214-3p axis and decreasing GPX4 [109], thereby promoting the ferroptosis, both in vitro and in vivo. However, due to the possibility of abuse of ketamine, it is may not available for clinical use yet. In addition to drugs, nanotechnology is also being used in the treatment of tumors. Luo Y et al. used nanoparticle-coated miR-101-3p to target TBLR1, promoting ferroptosis and inhibiting lung cancer progression [110]. These indicate that miRNAs have promising prospects in future clinical diagnosis and treatment (Table 3).

**Table 3:** Application of ferroptosis-related miRNAs in tumor therapy.

miRNA	Disposes	Cancer types	Expression levels in the sample	Models of evidence	References
miR-214	–	Prostate cancer	Downregulate	Clinical trials	[129]
miR-27a-3p	–	Prostate cancer	Upregulate	Clinical trials	[130]
miR-9	–	Nasopharyngeal carcinoma	Downregulate	Clinical trials	[131]
miR-17-92	–	Gastric cancer	Upregulate	Clinical trials	[132]
miR-375	–	Hepatocellular carcinoma	Downregulate	Clinical trials	[133]
miR-324-3p	–	Hepatocellular carcinoma	Downregulate	Clinical trials	[134]
miR-375	–	Colorectal carcinoma	Downregulate	Clinical trials	[135]
miR-137	–	Glioblastoma	Downregulate	Clinical trials	[136]
miR-324-3p	Icariside II	RCC	Upregulate	Cell culture	[108]
miR-214-3p	Ketamine	Liver cancer	Upregulate	Cell culture, animal models	[109]
miR-101-3p	Nanoparticle-coated	Lung cancer	Upregulate	Cell culture, animal models	[110]
miR-21-3p	Nanoparticle-coated	Melanoma	Upregulate	Cell culture, animal models	[137]
miR-744-5p	Propofol	NSCLC	Upregulate	Cell culture, animal models	[138]

Some ferroptosis-related miRNAs have been proved to be diagnostic or prognostic biomarkers of cancer and can be used for targeted therapy.

## Conclusion

Current basic research on ferroptosis is growing exponentially. miRNA is thought to play a different regulatory role in the ferroptosis process, but there are still some problems in this field. With too few existing studies, the specific mechanisms by which ferroptosis regulates the tumor metastasis process are still being explored. Moreover, although it is relatively clear that miRNA regulates ferroptosis and tumor metastasis, respectively, it is ambiguous how miRNA regulates tumor metastasis through ferroptosis. Nonetheless, we illustrate the new role of ferroptosis-related miRNA on the regulation of tumor metastasis, and the potential link between miRNA, ferroptosis, and tumor metastasis, thus providing new perspectives and thoughts for future experimental research. In the future, further extensive exploration of the relationship of miRNA in tumor metastasis and ferroptosis is needed, and it provides new targets and directions for the treatment of cancer by developing appropriate model systems to help in the diagnosis, treatment, and prognosis of cancer.
